# A New Approach to Measuring Student–Teacher Relationship in Dental Education: Validation of the Working Alliance Inventory for Education (WAI-EDU)

**DOI:** 10.1007/s40670-025-02306-x

**Published:** 2025-02-05

**Authors:** Gerhard Schmalz, Stefan Büchi, Dirk Ziebolz, Deborah Kreher, Thomas Gerhard Wolf, Daniela Ackermann-Piek

**Affiliations:** 1 Department of Conservative Dentistry and Periodontology, Brandenburg Medical School (MHB), Theodor Fontane, Geschwister-Scholl-Straße 36, 14776 Brandenburg an Der Havel, Germany; 2 Medix Gruppenpraxis, Zurich, Switzerland; 3 Department of General Medicine, Leipzig University, Leipzig, Germany; 4 Department of Restorative, Preventive and Pediatric Dentistry, School of Dental Medicine, University of Bern, Bern, Switzerland; 5 Department of Periodontology and Operative Dentistry, University Medical Center of the Johannes Gutenberg-University Mainz, Mainz, Germany; 6 SRH Fernhochschule – The Mobile University, Riedlingen, Germany

**Keywords:** Dental education, Undergraduate education, Self-reflection, Relationship, instrument validation

## Abstract

**Background:**

This study aimed at an initial validation of the Working Alliance Inventory for Education (WAI-EDU), an instrument to assess student–teacher relationship (STR).

**Methods:**

The WAI-EDU was derived from the original Working Alliance Inventory to fit the context of higher education. Following a pre-test, the tool was applied to two different study settings. In the first study part, 81 clinical students completed the WAI-EDU at baseline, after 1 h (t1), and after 1 w (t2), to assess reliability. For comparison, a visual analogue scale (VAS) was also used to evaluate STR. In the second study part, 60 preclinical students completed the WAI-EDU at baseline and at a follow-up after 1 week of intensive education. Cronbach’s *α*, Pearson correlation coefficient, and Mann–Whitney *U* test were used for statistical analysis.

**Results:**

Cronbach’s *α* of the WAI-EDU was higher than 0.9 at each time point in the cohort of 81 students. The test–retest-reliability was highest between baseline and t1 (*r* = 0.928). Even between baseline and t2, and between t1 and t2, values higher than *r* = 0.8 were determined. In addition, the WAI-EDU correlated with VAS scales for strength (*r* = 0.627, *p* < 0.01) and quality of STR (*r* = 0.596, *p* < 0.01). After 1 week of teaching in the second cohort (*n* = 60), a significantly higher WAI-EDU sum score was revealed (47.43 ± 7.46 vs. 40.73 ± 8.08, *p* < 0.01). During 1 week of education, similar to the WAI-EDU results, the VAS values for STR increased significantly (*p* < 0.01).

**Conclusion:**

The WAI-EDU demonstrated robust reliability and validity in measuring STR. These findings suggest that the WAI-EDU is a promising tool for evaluating STR.

**Supplementary Information:**

The online version contains supplementary material available at 10.1007/s40670-025-02306-x.

## Background

The student–teacher relationship (STR) appears to be crucial in higher education, as it is related to the outcome of learning processes and the professional socialization of the students [1]. A questionnaire-based study on 264 medical students in Pakistan revealed that students perceive the STR to be highly relevant in the context of stress and coping strategies [2]. Qualitative research supported this, highlighting that an appropriate STR influences well-being of both students and teachers [3]. In first year medical students, a mentoring program helped to develop a strong STR, supporting both academic performance and emotional well-being [4]. This underlines the importance of STR beyond academic achievement as an appropriate health- and well-being–related factor, although the evidence in higher education is still limited. Therefore, a meaningful aim in medical education appears a dialogue between students and teachers to build up a trustful relationship between them [5]. However, there is a power dynamic between students and teachers, which influences the STR [6]. Overall, STR appears highly important for medical education and overall higher education as well.

Against this background, an assessment of STR seems of interest in an education setting, to reveal the quality and strength of the relationship. Thus, several studies aimed to measure the STR in a medical teaching context. A Saudi Arabian study used a questionnaire with different items on STR, which was answered on a 4-point scale, showing that students perceive a good STR in a medical school [7]. One measurement to evaluate STR has already been described and is primarily developed in the primary and secondary school contexts: the student–teacher relationship scale [8]. Meanwhile, different forms of this scale, including a 14-item short form, have been developed and validated [9]. However, this tool is a rubric instrument that assesses the teacher’s perspective. One Chinese study elaborated a modified version of this scale for the college setting [10]. In the private college setting, the modified tool showed good reliability and validity, suggesting a promising approach to measure STR in higher education [10].

Nonetheless, knowledge of STR in dental and medical education is limited, which is why valid instruments to evaluate STR are still not available, especially from the students’ perspective. Such an instrument can help teachers and students to assess and reflect on their relationship, which could support academic work, progress, and learning success in a trustful environment. Therefore, this current study aimed at the development and first validation of a novel instrument to measure STR in dental education based on correct methodological standards for validating new instruments [11, 12], the Working Alliance Inventory for Education (WAI-EDU), a modified version of the Working Alliance Inventory [13]. For this purpose, two study parts were designed to evaluate internal reliability, test–retest reliability, and validity of the measurement. The basis for the instrument is a modified version of the working-alliance inventory, which has primarily been developed to measure the relationship between patient and therapist in the context of psychotherapy [13]. Thus, the original WAI was composed for the patient-therapist context and ambulant and stationary psychotherapy, to reflect relationship strength in the three domains bond, task, and goals. The instrument helps to monitor therapeutic processes and evaluate therapeutic measures during psychotherapy [11, 14]. Of course, the teaching setting in dental education differs from this original idea; teaching is not a “therapeutic” process and lectures are not “therapeutic measures.” However, the bond between students and teachers, and the joint work on tasks and goals are similarities. The overall settings also differ between original WAI and WAI-EDU because therapy is often a close and intimate situation, while teaching is often performed in group settings. Accordingly, the performance of the developed WAI-EDU version appears of high interest, while it seems questionable in what way those limitations affect the usability of the tool.

## Methods

### Study Design

The current investigation was reviewed and approved by the Ethics Committee of the Medical Faculty at the University of Leipzig in Germany (No: 117/20-ek). All participants were informed verbally and in written form and gave their written informed consent for participation. The evaluation of the Working Alliance Inventory for Education (WAI-EDU) consisted of two prospective, observational study parts in two different cohorts of participants.

### Sample

For this investigation, two cohorts of undergraduate dental students were recruited. For the first part, i.e., testing the reliability of WAI-EDU, a group of 81 students in the clinical years of undergraduate dental studies (fourth and fifth year) were included.

In- and exclusion criteria were as follows:

- Inclusion criteria: (1) Enrollment in the fourth or fifth year of the dental program at Leipzig University; (2) previously performed teaching lessons with the respective teacher, on which the WAI-EDU should be focused in the current study (G.S.); and (3) voluntary participation.
- Exclusion criteria: (1) Lack of prior participation in teaching lessons at Leipzig University (Germany) (e.g., students who transferred from another university); and (2) direct collaboration with the respective teacher being evaluated using the WAI-EDU (e.g., involvement in a doctoral thesis with the teacher).

For the second part, the WAI-EDU was tested during 1 week of education. For this, a group of 60 preclinical dental students in their first or second year of studies were recruited.

- Inclusion criteria were similar to the other study part: (1) Enrollment in the first or second year of the dental program at Leipzig University (Germany); (2) participation in a preventive dentistry course at Leipzig University (Germany); and (3) voluntary participation.
- Exclusion criteria were equal as for the first study part, with an additional exclusion of students, who repeated the course due to failure.

#### WAI-EDU

The WAI-EDU was composed based on the original WAI, which originally evaluated the therapeutic relationship between patients and their therapists [13]. Therefore, modifications were performed to adapt the questionnaire for the dental teaching context. For example, in some questions, “my therapist” was replaced by “my teacher.” Based on the respective questions, more or less comprehensive modifications were made. Afterward, the WAI-EDU was pre-tested with a group of ten dental students, to ensure understandability and appropriate wording. Minor modifications of the questions were discussed within the author group. The final WAI-EDU is attached as supplementary file 1.

### Visual Analogue Scale (VAS)

**Fig. 1:**
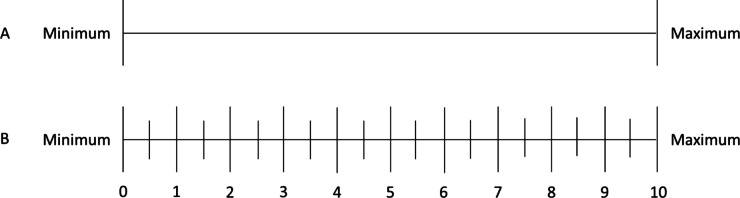
Example of the Visual Analogue Scale (VAS) (**A**) and the respective measurement tool (**B**). Thereby, the students only received scale A without numbers and sub-division, while B was used to translate the VAS into a numerical value. The respective questions were for example “How strong do you perceive your relationship with teacher XY between minimum, i.e. no relationship at all or inappropriate and maximum, i.e. an ideal strength of relationship?”

As a reference for testing the validity of the WAI-EDU, a visual analogue scale (VAS) was prepared. This VAS assessed the strength and the quality of STR. The example of a respective VAS is shown in Fig. 1.

### Study Flow

**Fig. 2:**
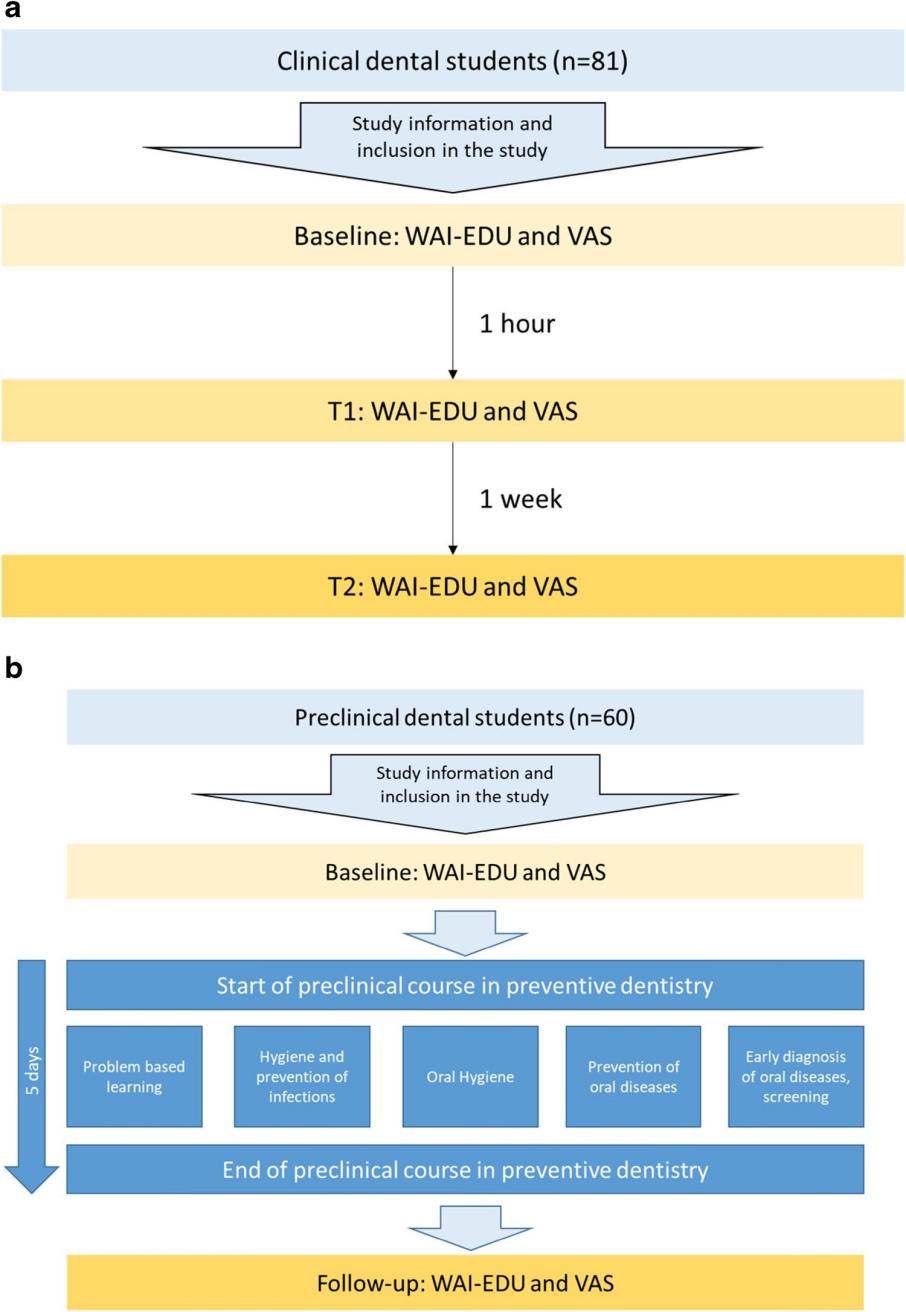
**a** Study flow for testing the reliability of the WAI-EDU in the cohort of clinical dental students. **b** Study flow for testing the WAI-EDU during a preclinical course in preventive dentistry. The teacher, who was used for the WAI-EDU evaluation, taught all of the elements during the week. He was supported by a team that only supported the practical part of the course

Two study parts were performed. In the first part, a group of 81 clinical dental students were surveyed using the WAI-EDU once at a baseline time point (independent of any teaching events), after 1 h and after 1 week, without any teaching events with the respective teacher in the meantime. Thereby, no teaching intervention was performed between baseline and the subsequent time points to evaluate the stability of questionnaire findings. At the same time points, the VAS was completed. The flow of this study part is shown in Fig. 2a.

The second part included a group of 60 preclinical students over 1 week of education in the form of a practical preclinical course in preventive dentistry. This preclinical course consisted of different thematic blocks during one week (4 h each day), whereby different forms of lectures were implemented. At the beginning and at the end of the week, a problem-based learning (PBL) was applied. Each day, a theoretic lecture in a seminaristic form was hold. During practical training, students performed oral hygiene instructions, measurements of oral hygiene, and professional tooth cleaning. Thereby, the WAI-EDU was completed, alongside with the VAS, at the beginning (baseline, before the course onset) and at the end of a practical course (follow-up, after 1 week of education). The course included intensive teaching with the teacher the WAI-EDU was tested for. This teacher performed all PBL sessions, seminars, and supervision of practical training. The students knew this professor prior to the week of education from a regular theoretical lecture in the first term of dental study. He was supported by a team, which co-supervised the practical training. The flow of this second study part is illustrated in Fig. 2b.

### Statistical Analysis

The statistical analysis has been performed with SPSS for Windows, version 25.0 (SPSS Inc., Chicago, IL, USA). Internal reliability was tested by Cronbach’s alpha. The test–retest reliability was determined by Pearson correlation analysis between the time points. Thereby, the reliability is based on correlation coefficients between 0 and 1 with the following interpretation: 0–0.1 = negligible correlation, 0.1–0.39 = weak correlation, 0.4–0.69 = moderate correlation, 0.7–0.89 = strong correlation, and 0.9–1 = very strong correlation [15]. For analysis and comparisons of the findings, sum scores were used. Thus, only completed questionnaires, i.e., those in which students answered all of the 12 WAI-EDU questions, were considered for analysis. The comparison between baseline and follow-up in the second part was performed using a Wilcoxon test. Prior to this, normal distribution was checked by the Kolmogorov–Smirnov test, showing non-normal distribution of the sample. All tests included a two-sided significance testing, with a significance level of *p* < 0.05.

## Results

### Reliability of WAI-EDU (Clinical Students in the Fourth or Fifth Year of Study, *n* = 81)

Table 1 shows the results of the internal reliability (Cronbach’s *α*) of the WAI-EDU, showing that the value was higher than 0.9 at each time point in the cohort of clinical students (*n* = 81, baseline, t1 (after 1 h) and t2 (after 1 week); Table 1).

**Table 1:** Internal reliability (statistical analysis: Cronbach’s *α*) of the WAI-EDU at three different time points in the cohort of clinical students (*n* = 81)

Time point	Cronbach’s *α*
Baseline	0.904
T1 (1 h after baseline)	0.945
T2 (1 week after baseline)	0.939

As shown in Table 2, the test–retest reliability was highest between baseline and t1 (*r* = 0.928). Even between baseline and t2, and between t1 and t2, values higher than* r* = 0.8 were determined (Table 2).

**Table 2:** Test–retest-reliability of the WAI-EDU in the cohort of clinical students (*n* = 81) between the three time points. Analysis was performed with Pearson correlation analysis

Time point	*r*	*p*-value
Baseline — T1	0.928	< 0.01
Baseline — T2	0.849	< 0.01
T1 — T2	0.879	< 0.01

In addition, the WAI-EDU moderately correlated with VAS scales for strength of STR (*r* = 0.627, *p* < 0.01) and quality of STR (*r* = 0.596, *p* < 0.01), while no significant correlations were found for the relevance of STR for stress, health, and academic performance (Table 3).

**Table 3:** Correlations between WAI-EDU and Visual Analogue Scale (*VAS*) questions in the total cohort of clinical students at baseline (*n* = 81). The correlation was tested by Pearson correlation analysis

VAS-Question	*r*	*p*-value
Strength of STR	0.627	< 0.01
Quality of STR	0.596	< 0.01
**Relevance of STR for …**		
Stress management	0.055	0.62
Academic performance	0.091	0.42
Health	0.016	0.88

### WAI-EDU During 1 Week of Dental Education (Preclinical Students in the First or Second Year of Study, *n* = 60)

**Fig. 3:**
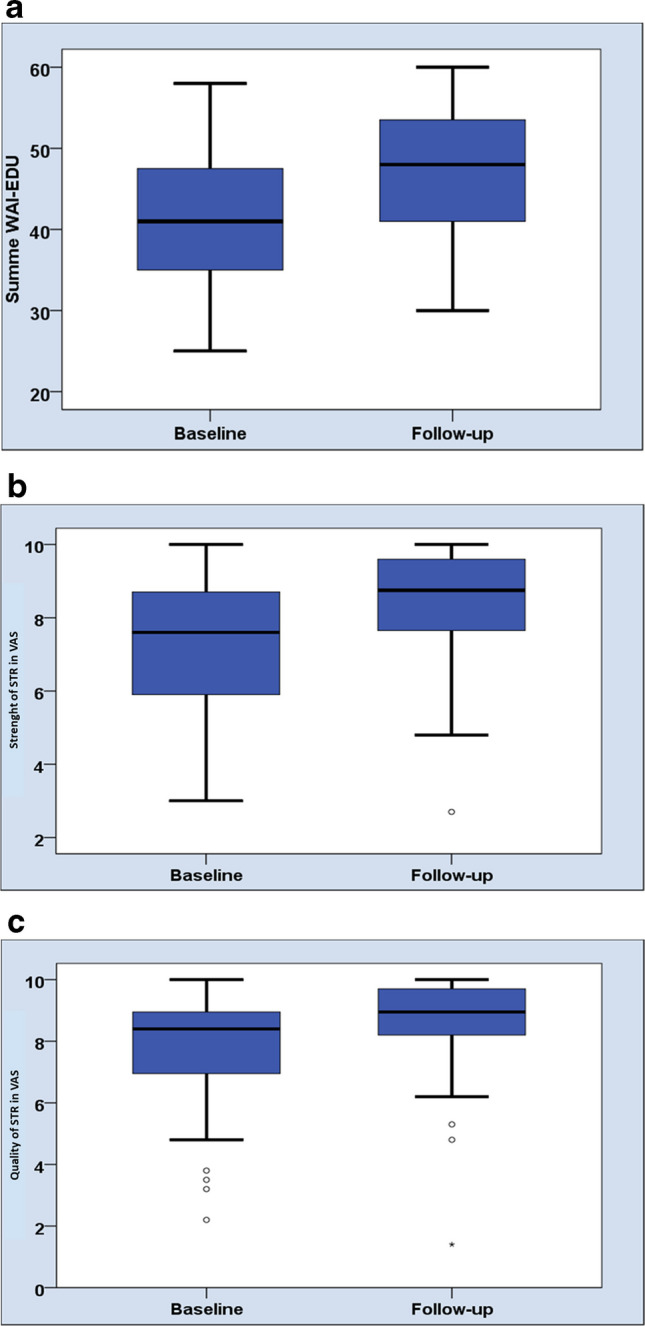
**a** Comparison of the mean sum score of WAI-EDU between baseline and follow-up (*p* < 0.01) in the cohort of preclinical dental students (*n* = 60). All participants completed the whole questionnaire at baseline and follow-up. **b** VAS for the question “How strong do you perceive the relationship with your teacher” between baseline and follow-up (*p* < 0.01). **c** VAS for the question “How do you perceive the quality of your relationship with your teacher” between baseline and follow-up (*p* < 0.01)

During 1 week of dental education, the WAI-EDU for the leading teacher of the intensive course in preventive dentistry increased. Thereby, a significantly higher sum score was revealed at follow-up (47.43 ± 7.46 vs. 40.73 ± 8.08, *p* < 0.01), as shown in Fig. 3a.

As shown in Table 4, all but one question of the WAI-EDU showed a significant increase between baseline and follow-up (*p* < 0.05). Only the question “My teacher and I respect each other.” did not change significantly (*p* = 0.07).

**Table 4:** WAI-EDU between baseline and follow-up in the cohort of preclinical students. Mean values and standard deviations are presented for all of the 12 questions

WAI-EDU question	Baseline (*n* = 60)	Follow-up (*n* = 60)	*p*-value
1. As a result of the teaching, I am clearer as to how I might be able to develop in the field of study	3.23 ± 0.91	4.10 ± 0.77	< 0.01
2. What I am doing in the teaching lessons gives me new perspectives on my personal and technical development	3.43 ± 1.00	4.13 ± 1.00	< 0.01
3. I believe my teacher likes me	3.13 ± 1.07	3.68 ± 0.79	< 0.01
4. My teacher and I collaborate on setting learning goals for me	3.23 ± 1.13	4.03 ± 0.69	< 0.01
5. My teacher and I respect each other	4.18 ± 0.93	4.40 ± 0.69	0.07
6. My teacher and I are working towards mutually agreed upon goals	3.57 ± 1.11	4.15 ± 0.85	< 0.01
7. I feel that my teacher appreciates me	3.60 ± 1.12	3.90 ± 0.84	0.02
8. My teacher and I agree on what it is important for me to work on	3.20 ± 1.09	3.88 ± 0.88	< 0.01
9. I feel my teacher cares about me even when I do things that he/she does not approve of	2.82 ± 1.00	3.35 ± 1.12	< 0.01
10. I feel that the things I do in teaching lessons will help me to accomplish the study aims that I want	3.88 ± 0.98	4.13 ± 0.75	0.03
11. My teacher and I have established a good understanding of the kind of changes that would be good for me	2.63 ± 1.02	3.50 ± 0.89	< 0.01
12. I believe the way my teacher and I are working with my technical development is correct	3.82 ± 0.97	4.17 ± 0.69	< 0.01

During 1 week of education, similar to the WAI-EDU results, the VAS values for strength and quality of STR also increased significantly (*p* < 0.01, Fig. 3b and c).

## Discussion

### Main Findings

The WAI-EDU demonstrated good internal and test–retest reliability in this monocentric cohort of dental students, both after 1 h and after 1 week. The correlation between WAI-EDU scores and the VAS measures for STR strength and quality, as well as the increase in WAI-EDU sum score after 1 week of intensive education, suggests that the instrument has acceptable convergent validity. The standardized questionnaire format minimizes potential bias from the test administrator, ensuring objectivity in the evaluation process.

### Discussion of WAI-EDU and Its Validity

The current study developed and provided initial validation of the WAI-EDU for measuring STR in the context of dental education. To date, few studies explored the reliability of STR in higher education. Only one comparable study, which was conducted at a Chinese private college, reported Cronbach’s *α* of 0.94 for a modified STR scale [10]. Those findings closely align with the Cronbach’s *α* in the current study. A Cronbach’s *α* exceeding 0.9, as achieved by the WAI-EDU, is considered highly reliable; this is especially relevant in medical research and related areas, where stringent reliability is required [16, 17]. Notably, the original WAI, which is widely used in psychotherapy, demonstrates similar levels of reliability [13, 18]. However, the limitations noted in the clinical application of the original WAI, e.g., influence of time point, severity of underlying diseases, setting (ambulant vs. stationary, involvement of multiple therapists) and a bias regarding social desirability, should also be considered when interpreting the present findings [13, 18]. Overall, the current study’s results demonstrate that the WAI-EDU is a reliable tool for assessing STR within this specific cohort of dental students. Regarding validity, moderate correlations between the WAI-EDU sum scores and the VAS ratings support the instrument’s ability to measure STR in this cohort, in line with established guidelines for interpreting correlation coefficients [15]. The use of VAS as a comparative measure appears appropriate, as it has been successfully employed in other medical education contexts [19, 20]. Thus, the current findings indicate that the WAI-EDU has a reasonable degree of validity, as reflected by its association with VAS measures of STR quality and strength. However, reducing a complex construct like STR into two dimensions such as quantity and quality of relationship might be a vast simplification. For example, Bai et al. [10] identified six dimensions of STR, including trust, interaction, intimacy, care, approval, and comfort, suggesting a more nuanced understanding of STR may be warranted [10]. Further validations of WAI-EDU should consider integrating these broader dimensions to enhance the comprehensiveness of the tool.

The validity of the WAI-EDU is supported by the increase of the sum score after a week of intensive teaching, especially because the VAS values increased the same way. This suggests that WAI-EDU is capable of detecting fluctuations in STR over a short period of time. Interestingly, only the question “My teacher and I respect each other.” did not differ between baseline and follow-up. This might indicate that “respect” is a stable issue, which is not easily affected by teaching events, but rather by general aspects between students and teachers. Overall, the WAI-EDU offers a novel and promising approach for evaluating STR in dental education. Its potential application in other fields of higher education, particularly medical education, is also conceivable. The results of this study provide a solid basis for further research and broader use of the WAI-EDU across different educational contexts.

### Strengths and Limitations

This is the first study to develop and validate an instrument specifically designed to assess STR in higher education from the student’s perspective in the context of dental medicine. The study design was robust, including both a pre-test and two independent study phases, aligned with the research aims of the study. The instrument was thoughtfully adapted from an existing, validated measure and underwent iterative refinement based on pre-test feedback. However, there are several limitations that should be considered. Sample size and selection are crucial for drawing statistically robust conclusions, especially in validation studies [21]. Although the current samples appear reasonable, the sample size might be too small overall. Especially, the monocentric setting limits the generalizability of the findings. Moreover, evaluating the validity with VAS and changes over time not only appears plausible, but is also limited in its robustness. Multicentric studies involving larger sample size and longer follow-up periods, potentially during a complete course or term (both clinical and pre-clinical), are needed to support the current findings to confirm the reliability and validity of the WAI-EDU. Furthermore, the current study assessed the STR with only a single teacher. One potential limitation is that the students knew him from a theoretical lecture in the previous term. During the 1 week of preclinical course, this teacher had the main amount and responsibility of the teaching events, but was supported by a team, especially in the practical elements. This should be emphasized in the interpretation of results. It is known from the original WAI in therapeutic context that multiple therapists in stationary setting might negatively affect the results [13]. However, especially in a practical dental course, more than one teacher is usually involved, making the setting in the current study quite close to teaching reality. By now, it remains somewhat questionable, how adaptable the WAI-EDU would be to a team-teaching situation that is more normal in medical education compared with a single therapist that is more typical of the scenario for which the original WAI was designed for. This should be emphasized for future research in the field. Expanding the validation of the instrument to include multiple instructors and varying educational contexts with a differentiation between theoretical courses, practicals, or tutorials could be a relevant addition. Thus, further research will be needed to finally validate the instrument WAI-EDU for higher education across different samples and contexts.

### Potential Implication for Teaching Practice and Future Perspective

The available literature clearly depicts STR to be of relevance in higher education; Hagenauer et al. (2023) summarized that a positive STR would be important for well-being, teaching quality, and learning process [22]. Earlier, Haidet and Stein stated STR to be part of the “hidden curriculum” in the formation of physicians [23]. In this previous work, it has already been noted that data or information on the STR are needed for educators to understand the educational process, respectively [23]. The WAI-EDU might be able to provide such information on a quantitative basis. Especially in the early years of studies, STR appears important as it helps to enhance confidence and makes the transition easier for the students [24]. The WAI-EDU could help to quantify the STR in this early stage of studies, providing feedback for the teachers regarding this issue. On this basis, teachers could identify inappropriate STR and its dimension based on the single WAI-EDU question and would have a basis to work on. Subsequently, the WAI-EDU would deliver quantitative information on a positive (or negative) progress during follow-up, as it appears sensitive against changes. Nevertheless, this concept relies on the willingness of teachers and faculties to evaluate the STR and to invest in its improvement.

Against the literature, the usage of WAI-EDU in the current study appears still limited as it only provides the student perspective. However, the teacher-perspective is also relevant in context of STR [22]. Therefore, the concept of WAI-EDU might be extended by developing a teacher-version for the assessment of both perspectives. Overall, the quantification of STR using WAI-EDU would bring a measurable insight for faculty members, which might offer relevant information to foster professional and personal development of students. However, further validation and evaluation of the instrument will be needed to confirm the upper mentioned conceptual ideas and to reveal the usability of WAI-EDU in higher education.

## Conclusion

Within the limitations of this monocentric initial validation study, the WAI-EDU was found to be a reliable, valid, and objective tool to measure STR in undergraduate dental students. The WAI-EDU can be seen as a promising tool to evaluate STR in higher education, but further large-scale multicentric validation is required to confirm the results across different samples and contexts.

## Supplementary Information

Below is the link to the electronic supplementary material.

Supplementary file1 (DOCX 37 KB)

## Data Availability

The datasets used and/or analyzed during the current study are available from the corresponding author on reasonable request. The data are not publicly available because of the pseudonymization and data protection guidelines according to the ethics approval.

## Abbreviations

STR: Student-teacher relationship
VAS: Visual analogue scale
WAI-EDU: Working Alliance Inventory in Education
