# Supplementary material for: A New Approach to Measuring Student–Teacher Relationship in Dental Education: Validation of the Working Alliance Inventory for Education (WAI-EDU)
Source: Med Sci Educ. 2025 Feb 5;35(3):1323–31. doi: 10.1007/s40670-025-02306-x (PMC12228614; doi:10.1007/s40670-025-02306-x)
Supplement: Supplementary file 1 — Supplementary file1 (DOCX 37 KB) [file 40670_2025_2306_MOESM1_ESM.docx]

**Supplementary Information**

**Supplementary material**

**Supplementary figure 1.** WAI-EDU in the applied form in this current study.

|  | seldom | sometimes | fairly often | very often | always |
| --- | --- | --- | --- | --- | --- |
| 1. As a result of the teaching I am clearer as to how I might be able to develop in the field of study. | 1 | 2 | 3 | 4 | 5 |
| 1. What I am doing in the teaching lessons gives me new perspectives on my personal and technical development. | 1 | 2 | 3 | 4 | 5 |
| 1. I believe my teacher likes me. | 1 | 2 | 3 | 4 | 5 |
| 1. My teacher and I collaborate on setting learning goals for me. | 1 | 2 | 3 | 4 | 5 |
| 1. My teacher and I respect each other. | 1 | 2 | 3 | 4 | 5 |
| 1. My teacher and I are working towards mutually agreed upon goals. | 1 | 2 | 3 | 4 | 5 |
| 1. I feel that my teacher appreciates me. | 1 | 2 | 3 | 4 | 5 |
| 1. My teacher and I agree on what it is important for me to work on. | 1 | 2 | 3 | 4 | 5 |
| 1. I feel my teacher cares about me even when I do things that he/she does not approve of. | 1 | 2 | 3 | 4 | 5 |
| 1. I feel that the things I do in teaching lessons will help me to accomplish the study aims that I want. | 1 | 2 | 3 | 4 | 5 |
| 1. My teacher and I have established a good understanding of the kind of changes that would be good for me. | 1 | 2 | 3 | 4 | 5 |
| 1. I believe the way my teacher and I are working with my technical development is correct. | 1 | 2 | 3 | 4 | 5 |
